# Intersecting SARS-CoV-2 spike mutations and global vaccine efficacy against COVID-19

**DOI:** 10.3389/fimmu.2025.1435873

**Published:** 2025-03-07

**Authors:** Samaneh Tokhanbigli, Samira Salami Ghaleh, Karim Rahimian, Mohammadamin Mahmanzar, Saleha Bayat, Shahrzad Ahangarzadeh, Bahman Moradi, Reza Mahmanzar, Yunliang Wang, Brian Gregory George Oliver, Youping Deng

**Affiliations:** ^1^ School of Life Sciences, Faculty of Science, University of Technology Sydney, Sydney, NSW, Australia; ^2^ Department of Computer Science, University of Tabriz, Tabriz, Iran; ^3^ Institute of Biochemistry and Biophysics (IBB), University of Tehran, Tehran, Iran; ^4^ Department of Quantitative Health Sciences, John A. Burns School of Medicine, University of Hawaii at Manoa, Honolulu, HI, United States; ^5^ Department of Biology and Research Center for Animal Development Applied Biology, Mashhad Branch, Islamic Azad University, Mashhad, Iran; ^6^ Infectious Diseases and Tropical Medicine Research Center, Isfahan University of Medical Sciences, Isfahan, Iran; ^7^ Department of Biology, Faculty of Sciences, Shahid Bahonar University of Kerman, Kerman, Iran; ^8^ Department of Biology, Science and Research Branch, Islamic Azad University, Tehran, Iran; ^9^ Department of Neurology, The Second Affiliated Hospital, Zhengzhou University, Zhengzhou, Henan, China; ^10^ Respiratory Cellular and Molecular Biology, Woolcock Institute of Medical Research, Sydney, NSW, Australia

**Keywords:** D614G, P681, E484, Y655, mutation, SARS-CoV-2, spike, vaccination

## Abstract

In line with encountering the world with the emergence of vaccine-resistance variants of SARS-CoV-2, 15,669,529 samples that received COVID-19 vaccines until April 2023 were investigated as two doses in the first phase and booster vaccinations in the second phase. The analysis shows that D614G and P681 mutations occurred in both phases. The E484 and Y655 mutations significantly emerged during the second phase. The 762-889 and 254-381 regions are revealed as conserved parts and could be considered in vaccine design. The Kruskal–Wallis test revealed a significant reduction in single mutations between populations with 20%–50% and those with 70%–100% vaccination coverage (p=0.017). The Mann–Whitney U test proposes a link between vaccination and suppression of viral mutation rates. Dynamic modeling suggests that key mutations have facilitated the virus’ evolution and immune escape. The study’s findings are crucial for understanding virus genome mutations, especially E614 and P681 in Delta and E484 and H655 in Omicron. This highlights the need to adjust strategies and strengthen global efforts in combating the pandemic.

## Introduction

The World Health Organization (WHO) declared a pandemic due to the novel severe acute respiratory coronavirus 2 (SARS-CoV-2) infection in early 2020 and named it coronavirus infection disease 19 (COVID-19). The virus, similar to SARS-COV, belongs to the beta-lineage coronaviruses. The encapsulated single-stranded RNA of the virus encodes several non-structural proteins and approximately 30 functional proteins (1–3). The spike is critically essential in infection and immunogenicity mediating virus entry into cells (4). Immediately after binding the spike to the cell receptor, two proteases, furin and TMPRSS2, cleavage the spike’s S1/S2 and S2 subunits, respectively (5). The first cleavage exposes the receptor-binding domain (RBD) and/or the S2 subunit after disassociation and shedding of S1. The proteolytic activity of TMPRSS2 results in the S2 subunit cleavage, the binding of virus capsid to the cell membrane, and the infusion of virus RNA to the host cell (6). Neutralizing antibodies (nAbs) induced by vaccines are the cornerstone of COVID-19 treatment (7). Various vaccines were developed, many based on the spike protein (8), particularly the S1 subunit, which contains the neutralizing antibody epitopes mainly on the RBD (located on the C-terminus of S1) and the recognition sites (9). However, the fast transmission of the virus and the emergence of various mutations due to amino acid (A.A) changes in the spike region obligate monitoring to improve the vaccine design. For instance, the mortality rate of SARS-CoV-2 in some countries was associated with the dominance of D614G mutation in the spike protein and various variants such as delta and omicron, even with the administration of booster vaccinations (10, 11). On the other hand, the P681 mutation has a key role in the replacement of the Alpha-to-Delta variant (12). These mutations still exist in samples despite the vaccination’s upward trend. This issue emphasizes the critical role of these spike mutations in spreading the disease globally and subsequently affecting the efficacy of developed vaccines (13, 14). Furthermore, tracking these mutations in the spike protein during various phases of vaccine administration across countries could provide insights into the evolution of SARS-CoV-2 mutations and vaccine efficacy.

**Figure 1:**
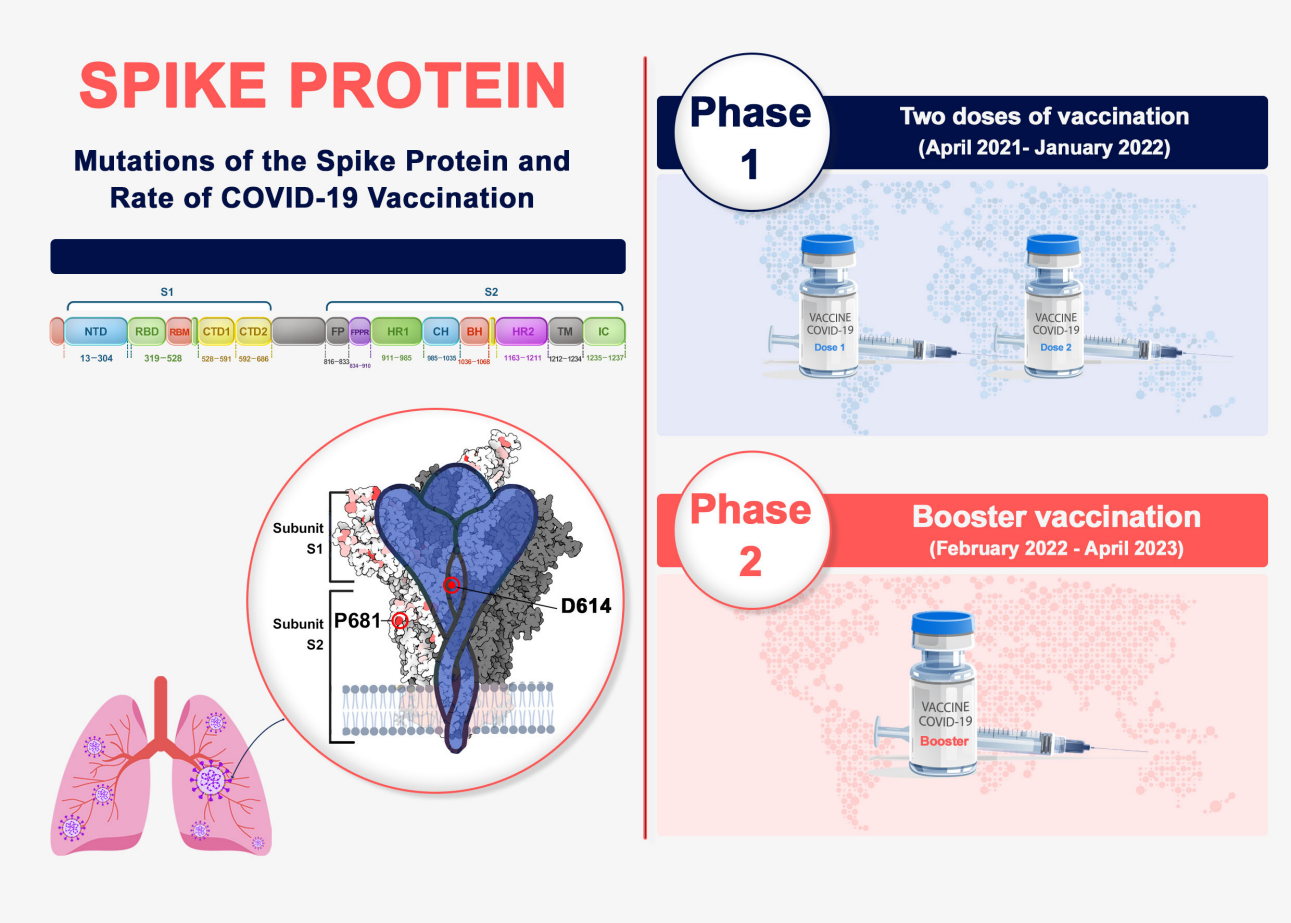
Spike protein genome and phases of vaccination in COVID-19. Spike genome organization includes two S1 and S2 subunits. The top mutation occurrences in spike were analyzed in two phases of vaccination progress.

In this study, we aim to monitor the spike protein’s amino acid mutation pattern using the GISAID and Sars2Mutant databases (15, 16). We will compare the rate and type of mutations between the two vaccine doses and booster vaccinations in countries where more than 50% of the population has received booster shots. As a result, a big data approach by the Sars2Mutant database (17) is used to look for variants, as well as these A.A mutation patterns of spike protein in these time points. The findings provide valuable information supporting the design of more effective treatment, primarily vaccines for COVID-19 (**Figure 1**).

## Methods

### Data repositories and spike variant calling

The present study was designed to address the mutations as a reflection of genome sequence in the spike protein of SARS-CoV-2 compromising 1,273 A.As during the different time points of vaccinations. The top A.A changes were chosen from countries that got 50% and more vaccination. These mutations in the spike region were investigated in specific eligible vaccinated countries categorized into two phases of vaccinated populations, which are presented as follows by exact periods:

• Phase 1 (first two doses of vaccination): At least 50% of the population had two doses of vaccination, as shown in **Supplementary Tables 1**, **2** (April 2021-January 2022)

○ Before vaccination (March 2020-December 2020)
○ Initial vaccination (January 2021-March 2021)
○ Middle of vaccination (April 2021-Jun 2021)
○ End of vaccination (July 2021-January 2022)

• Phase 2 (booster): More than 50% of people had booster vaccination, as shown in **Supplementary Table 3** (February 2022-April 2023)

Data analysis was carried out by trimming the outputs in a specific pattern. Within each phase, mutations were ranked based on their frequency across eligible countries, those meeting the vaccination coverage threshold. From this ranking list, the top four highest mutation rates in multiple countries were selected. These four mutations exceeded the ≥50% prevalence threshold across all or the majority of eligible countries in that phase. Eventually, these top mutations were considered significant and frequent by rate of substitution to report as phase 1 and 2 results and then compare them.

Further metadata analysis was carried out by Python 3.8.0 to isolate the spike sequence. Furthermore, the FASTA sequences of the SARS-CoV-2 spike protein (S1 and S2 subunits) were aligned to the reference sequence, and the variants were called. Among the obtained records of spike protein sequences, trimming was carried out on the non-human samples, and sequences with less than 1,273 A.As and non-specified A.As were selected. Finally, 15,669,529 sequences were included in the current study. The “Numpy” and “Pandas” library approaches were adopted to improve the efficiency of all stages. The applied algorithm for identifying the mutations is described as follows. Since all sequences have equal lengths, the following algorithm used “Refseq” and “seq” to refer to the reference sequence sample sequence, respectively.

*For refitem, seqitem in zipping (RefSeq, seq)*
*If (refitem! =seqitem)*
*Report a new mutant*

Subsequently, the determined mutations of SARS-CoV-2 locations were classified based on the continent, country names, and countries’ global coordinates using country-convert 0.5.8 software and the “Titlecase” library in Python.

The gathered data on vaccination based on the reports from the New York Times (https://www.nytimes.com/interactive/2021/world/covid-vaccinations-tracker), the economists (https://www.economist.com/graphic-detail/tracking-coronavirus-across-the-world), and our world in data (https://ourworldindata.org/covid-vaccinations). Furthermore, the countries underwent the trimming with the following criteria:

1. The recipient of booster vaccination was considered fully vaccinated and included in the study.
2. Countries have vaccinated their populations with similar vaccines in different groups (only FDA-approved vaccines, namely: Pfizer/BioNTech, Moderna, Johnson & Johnson, AstraZeneca, Sinovac, Sinopharm/BIBP, Covishield, Covaxin, and so on).
3. The registered sequences are normal and are analyzable.
4. Samples exclusion of: divergent, short, or lengthy sequences, gaps, unspecified amino acids (indicated by X), and genomes from non-human hosts.
5. Each country has equal input NGS data regarding the number of next generation sequence (NGS) data.

The initial strain of the COVID-19 virus, designated as “EPI_ISL_402124,” served as the benchmark reference sequence for aligning all subsequent samples. To explore and call variants, the GISAID (www.gisaid.org) data source was utilized to collect the data from July 2021 to April 2023 with 15,669,529 sequences (16, 18, 19). It is worth mentioning that trimming was applied based on the nonsense changes and drawing out the sequences with deletions, which resulted in a shorter sequence of 1,273 A.As. Access to GISAID was provided by permission of John A. Burns School of Medicine Department of Quantitative Health Sciences.

### Statistical analysis of mutations and vaccination stages

The quantitative counts of SARS-CoV-2 mutations were standardized, and statistical analyses, including the Kruskal–Wallis test and Mann–Whitney U test, were applied to ascertain differences in mutation counts across vaccination rate groups. Correlation coefficients were computed for pairwise phase comparisons, and two heatmaps were constructed to synthesize the findings. The study provided a detailed explanation for the selection of the statistical tests, highlighting their suitability for the non-parametric nature of the data, ordinal data, unequal sample sizes, and specific hypothesis testing. Ethical considerations were addressed, and institutional review board approval was not required as the study involved the analysis of publicly available data without direct human or animal subjects, and all methods were performed following relevant guidelines and regulations.

### Secondary protein structure and dynamic prediction

The study analyzed the mutational structure and molecular flexibility of the spike protein modeling on the D614G, E484K, and H655Y mutations, which are the most frequent mutations in COVID-19. The DynaMut web server (http://biosig.unimelb.edu.au/dynamut/) was used to perform the analysis, and the PDB ID of protein (7QUS) was taken from the Protein Data Bank (https://www.rcsb.org/).

## Results

To investigate the mutation dynamics in spike protein during the vaccinations, we carried out the mutational analysis. All the reported mutations were the identified mutations at the end of vaccination (July 2021-January 2022), highlighting their dominance and stability in the viral population.

**Figure 2:**
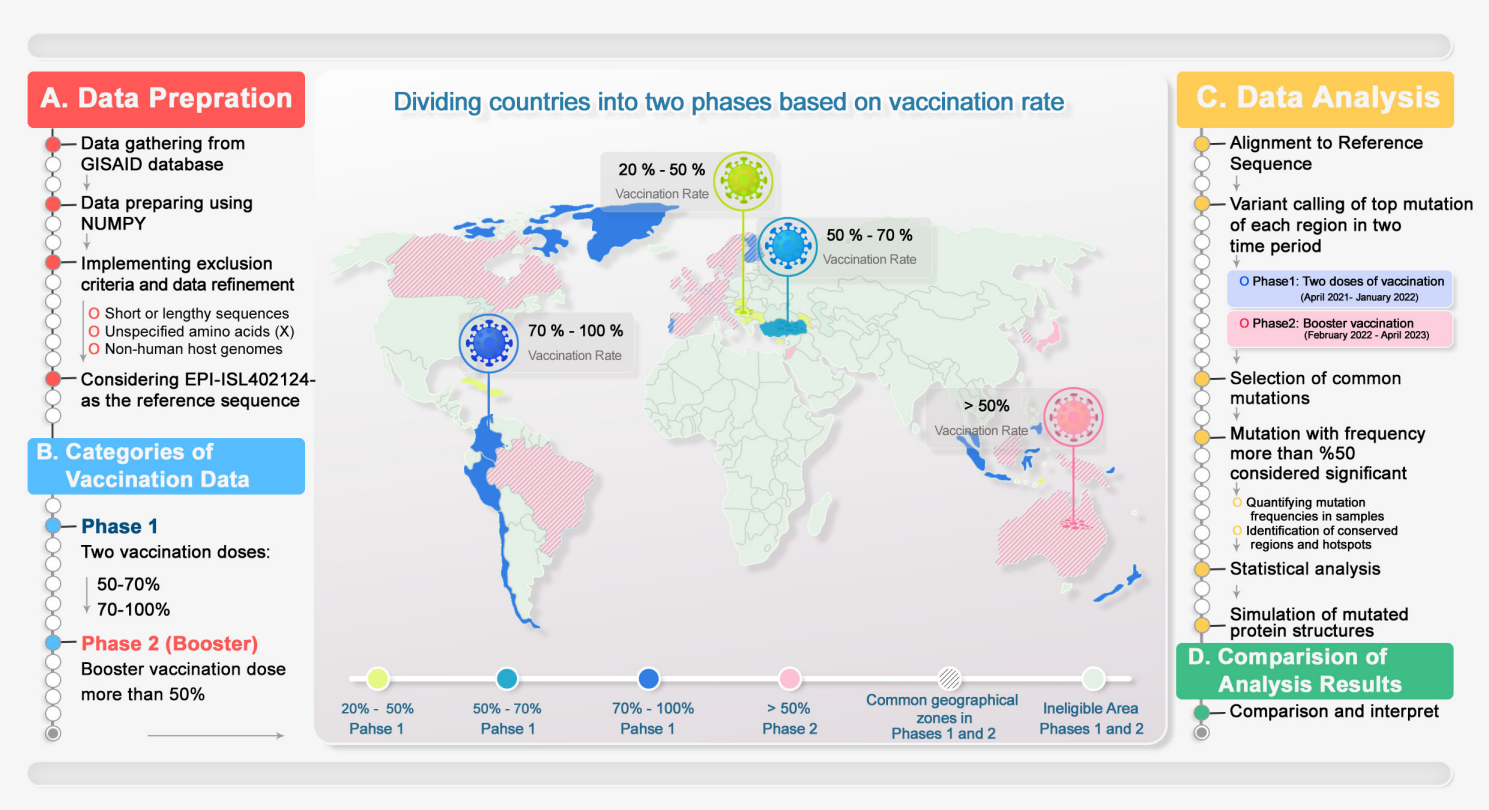
Methodology overview. This is a schematic representation of the data mining process, categorized into two distinct phases: data refining and analysis, followed by data reporting. Countries were categorized into two phases based on number of received doses of COVID-19 vaccines. Overlapping areas between groups were hatched. 1) Data from GISAID were extracted and analyzed, and the NGS data criteria for each country and group were applied. 2) Geographical data categorized based on doses of vaccination with a chance of 50% occurrence in each phase of vaccination were reported. 3) The Wuhan genome was considered the reference genome. Next, the obtained mutations were processed. The common mutations with more than 50% at the different vaccination phases were concluded. 4) The top mutations of each period of vaccination were compared, and common or unique mutations were presented to help the vaccine design industry.

These mutations were investigated during two distinctive phases (phase 1 receiving two doses of vaccination and phase 2 being the booster). The occurrence threshold was at least 50% in all countries (equal to more than 10 repeats). In this regard, the mutual mutations in each separated group are reported and compared. **Figure 2** illustrates the workflow from data gathering, processing, and analysis.

### Common mutation patterns across countries in two distinct phases

The top detected mutations in each phase at specified vaccination periods were selected, and then the mutual mutations were highlighted. Only the samples with more than 50% repetitive mutations in each phase were reported in the next step. All the common mutations in different phases are presented in **Table 1**.

**Table 1:** Mutations and vaccination in COVID-19: A comparative analysis of two phases.

Phase 1 (two-dose)						Phase 2 (booster)	
20%-50%		50%-70%		70%-100%		>50%	
Mutations	Frequency (%)	Mutations	Frequency (%)	Mutations	Frequency (%)	Mutations	Frequency (%)
D614G	99.98	D614G	99.96	D614D	99.95	D614	95.92
P681	99.23	T478K	92.41	P681	97.06	P681	94.69
R158G	88.28	L452R	92.28	R158G	95.03	E484	93.88
D950N	87.87	T19R	92.25	L452R	94.46	H655	93.27
L452R	86.42	P681	92.02	T478K	94.39		
T478K	86.4	R158G	91.71	D950N	94.15		
F157-	86.3	F157-	91.7	T19R	93.97		
E156-	86.25	E156-	91.66	F157-	93.03		
T19R	85.53	D950N	90.63	E156-	93		
G142D	82.05	G142D	76.28	G142D	82.27		

This table presents the top four mutations identified in the study investigating the relationship between mutations and vaccination in COVID-19. The study categorized the vaccination process into two phases based on the doses of vaccination—two doses of vaccination and booster dosage—and found that two of the top mutations were shown in each of the two phases, whereas two high-rate E484 and H655 were only reported after booster vaccination in the population.

#### Phase 1 two doses of vaccination and mutation patterns

##### Countries with 20%–50% vaccination coverage

The 20%–50% group in phase 1 (population receiving two doses of vaccine) was selected to investigate the initiation of mutation dynamics compared with the Wuhan genome. Furthermore, this phase of vaccination is considered as the baseline for before, initial, middle, and end periods presented in **Supplementary Table 4**.

This group covers the countries where only 20%–50% of their population have received two doses of their vaccines. P681H/R/- mutation was observed in the initial, middle, and end of the designated time points for a vaccination with (64.1%, 94.43%, and 99.23%) respectively. P681 mutation with A.A conversion to histidine was dominant during the initial (58.07%) and middle (88.1%); however, the deletion of the proline at the 681 positions was the prevalent substitution seen at the end of the selected vaccination time point with 78.73%. The mutations E156- (86.52%), F157- (86.3%), L452R (86.42%), T19R (85.53%), T478K (86.4%), D950N (87.87%), G142D (82.05%), and R158G (88.28%) were the other mutations detected only at the end of the selected time points for vaccination (**Table 1**).

##### Countries with 50%–70% vaccination coverage

In the countries that had 50%–70% of the population vaccinated, the most frequent mutation was D614G detected at the end of the phase 1 period of vaccination with a high rate of up to 99.96%. Furthermore, P681 is another mutation detected in all the stages of the phase 1. The P681 substitution mutation shows with a gradual increase toward the end of phase 1, reaching a prevalence of 92.02%. Considerably, we found D950N (90.63%), E156- (91.66%), F157- (91.7%), L452R (92.28%), R158G (91.71%), T19R (92.25%), T478K (92.41%), and G142D (76.28%) mutations at the end of the selected phase 1 vaccination period (**Table 1**).

##### Countries with 70%–100% vaccination coverage

In countries with coverage of 70%–100% vaccinated population in phase 1, the D614G mutation is the most common mutation among all of the top mutations that maintained itself as long as two doses of vaccination were ongoing at a high rate (99.95%). On the other hand, P681 was another common mutation during all the assigned time points of investigation, with a significant increase among the nations with 97.06% at the end of the first phase (**Supplementary Table 5**). Some of the mutations were noticed to be significant at the end of the phase 1 period of vaccination in these countries, such as E156- (93%), F157- (93.03%), L452R (94.46%), T19R (93.97%), T478K (94.39%), D950N (94.15%), R158G (95.03%), and G142D (82.27%) (**Table 1**).

### Comparative analysis between mutations in 50%–70% vs. 70%–100% vaccinated populations in phase 1

The performed analysis presented notable results about the maintenance of mutations in the spike protein. All frequent mutations in 50%–70% of the dose-vaccinated group were also detected in 70%–100% of the vaccinated population in phase 1. Despite continuous process of vaccination, the rate of each mutation has increased, which could be a useful sign in vaccine design industry.

#### Phase 2 insights: similarities and differences in countries with over 50% booster vaccination coverage

In countries with coverage of more than 50% booster, D614G was the most frequent mutation with a significant rate among the nations (95.92%). Also, it is notable that P681, E484, and H655 mutations were observed in all eligible countries along the booster vaccination dose with 94.69%, 93.88%, and 93.27%, respectively (**Table 1**).

#### Mutations across phases 1 and 2

After investigating separately into two phases of vaccination, we found the two common mutations in both two doses of vaccination and booster periods. D614G and P681 also appeared and were conserved in the second phase despite booster vaccination. However, we also discovered that the E484 and H655 mutations in the booster dosage are distinct variants that were not found in phase 1, which is a noteworthy finding that should be taken into account when designing vaccines.

### Variant calling rate in investigated samples in each group in the first phase of immunization and booster dose

#### Countries with 20%–50% two-dose vaccination

**Figure 3:**
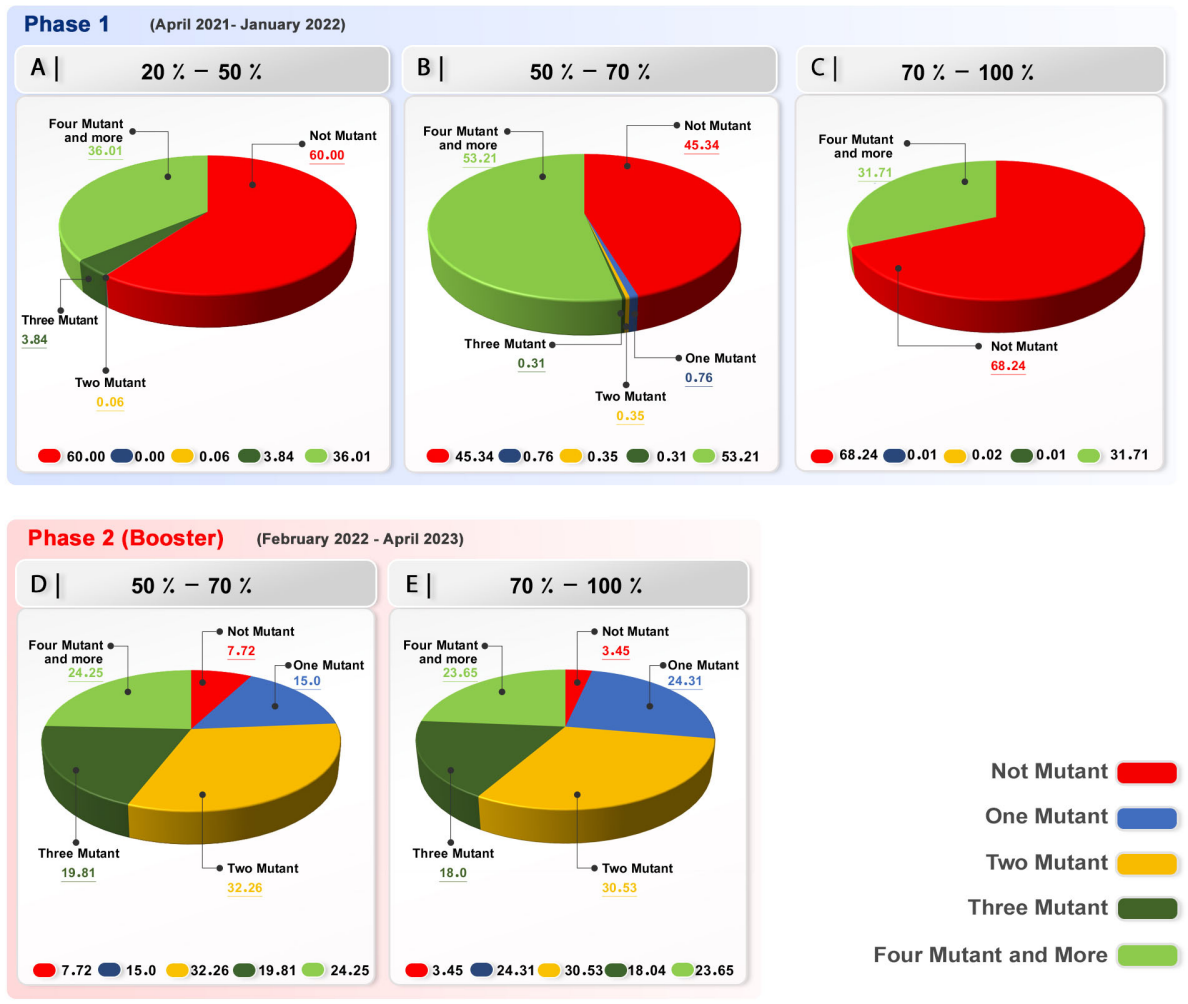
Variant calling rate in investigated samples in each group in the first phase of immunization and booster dose. Phase 1 **(A)** Group with 20-50% receiving two doses of vaccine; a significant number of sequences demonstrated no mutation in their spike. **(B)** Group with 50-70% receiving two vaccine doses; a significant number of sequences demonstrated no mutation in their spike. However, four mutations in the investigated samples were nearly equal to no mutation. **(C)** In the group with 70-100% two-dose-vaccinated population in phase 1, with the progress of the vaccination, a significant number of sequences demonstrated no mutation in their spike. Phase 2 (Booster) **(D, E)** Booster group, 50% of the population receiving booster dose; a significant number of sequences demonstrated more than one mutation in their spike.

This study group showed that, before the initiation of immunization, a single, dominant mutation existed, with a frequency of 46.62%. The majority of the investigated spikes (54.63%) had four or more alterations at the start of vaccination. With the progress of the vaccination, the four and more mutations in spike emerged and 52.62% and 31.63% of the sequences showed no mutation (**Figure 3A**; **Supplementary Table 4**).

#### Countries with 50%–70% two-dose

The number of mutations in this group can be categorized into two distinct sections. The first section shows that 51.87% of the spike sequences had no mutations, whereas the second section, comprising 46.12% of the sequences, had four or more mutations, indicating a significant increase after vaccination (**Figure 3B**; **Supplementary Table 1**).

#### Countries with 70%–100% two-dose

This group’s data, with a focus on the number of mutations in different vaccination stages, demonstrated that the population in phase 1 had a higher percentage of four or more mutations at the beginning of vaccination with 47.83%. Interestingly, at the end of vaccination, 70% or more of the population had received two doses of vaccine, and 63.7 of the samples showed no mutation in their spike. These findings highlight the efficiency and inhibitory effect of the vaccination when 70% or more of the population has been immunized (**Figure 3C**; **Supplementary Table 2**).

#### Phase 2 (booster dose)

The booster immunization phase in our study demonstrated that the occurrence of two mutations was dominant (31.23%). There were four or more mutations in the sequences, and one mutation was detected at 22.93% and another at 22.44%. Three mutations are present in the sequences of 18.08% of the samples (**Supplementary Table 3**). Nonetheless, a low percentage of the sequences (5.30%) displayed no mutation in their spike.

The result highlights the effect of vaccination on the mutation dynamic of the spike, and by increasing the number of vaccinated population and, more importantly, the fast rate of vaccination, the occurrence of mutation diminishes (**Figures 3D–E**).

### Vaccination impact: heatmap analysis of hotspots and conserved spike regions

**Figure 4:**
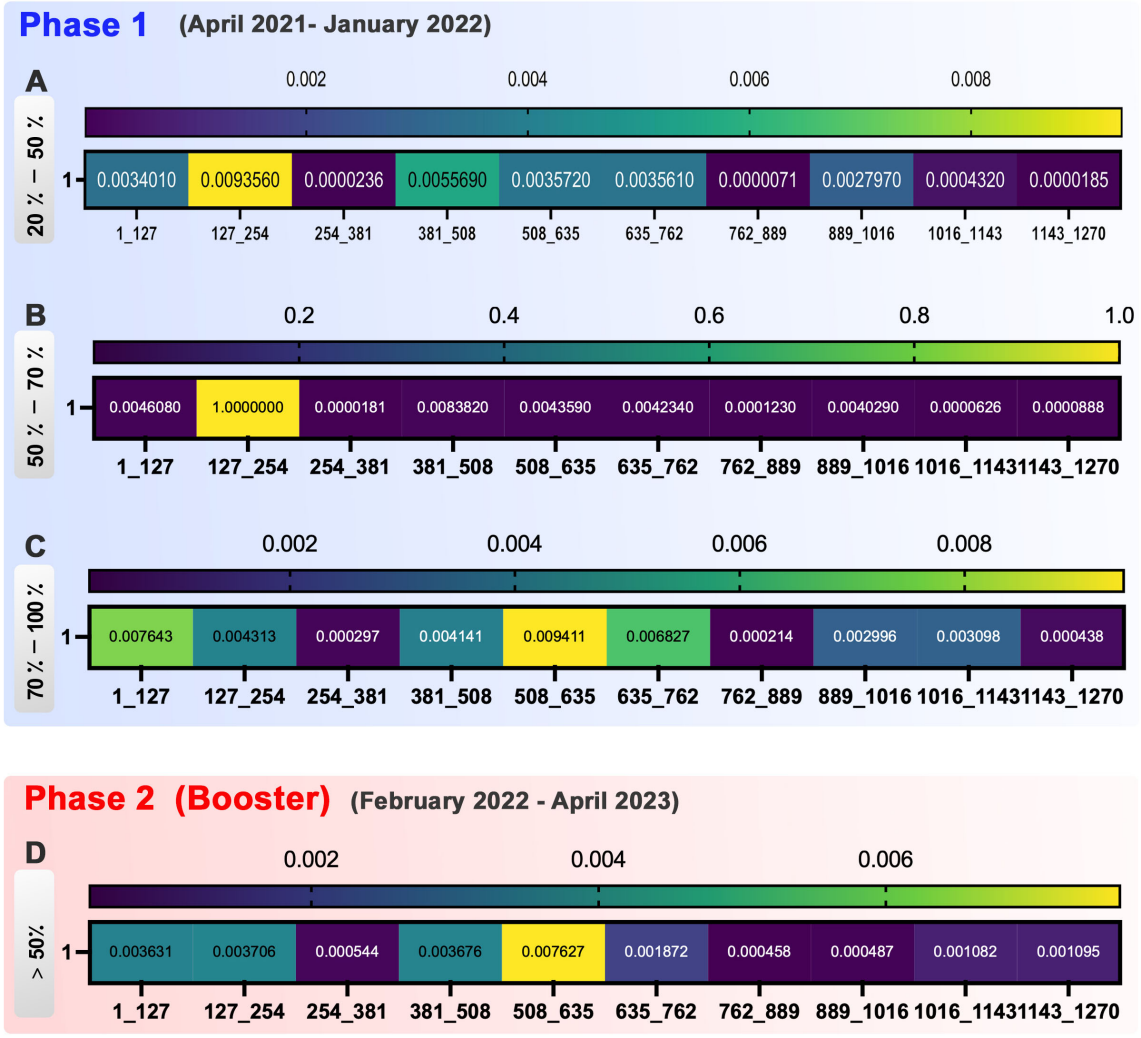
Heatmap represents the occurrence of spike mutations at various phases of vaccination. Phase 1, **(A)** In the 20-50% group of phase 1, 254-381, 762-889, and 1,016-1,270 were shown as conserved regions. **(B)** In the 50%-70% group of phase 1, the hot spots of mutation incidence are 127-254 regions in spike. Furthermore, 254-1,270 were detected as the conserved site. **(C)** In the 70%-100% group of phase 1, sequences 254-381, 762-889, and 1,143-1,270 are conserved to the mutation incidence, whereas 1-127, 381-508, 635-762, and 889-1,143 were more susceptible to mutations. Phase 2 (Booster), **(D)** In phase 2 of vaccination, the hot spots of mutation incidence are next to each other in this group as 381-762 regions. Besides them, 254-381 and 762-1,016 are shown as conserved regions.

We then categorized the spike sequence into 10 regions (1-127, 127-254, 254-381, 381-508, 508-635, 635-762, 762-889, 889-1016, 1,016-1,143, and 1,143-1,270), and we evaluated the mutation dynamics during the different periods of immunization to evaluate the susceptible parts of the spike to the mutations (**Figure 4**).

In group 20%-50% of phase 1, the dynamic of mutations showed high variation at the different time points. Sequences 254-381, 762-889, and 1,143-1,270 were the most conservatory sequences with minor mutation occurrence in this group (**Figure 4A**).

In the 50%-70% group of phase 1, sequence 127-254 demonstrated to be the hot spot for mutation occurrence by vaccination progress. The other hot spots of mutation detected in spike during the vaccination were the 381-508, 635-762, and 889-1,016 sequences. The conserved site was 254-381, with the lowest mutation incidence (**Figure 4B**).

In the 70-100% group of phase 1, the sequences 1-127, 381-508, 635-762, and 889-1,143 were more susceptible to mutations during the vaccination process. On the other hand, sequences 254-381, 762-889, and 1,143-1,270 simultaneously were conserved for mutation incidence (**Figure 4C**).

Despite the variation in mutation frequency in the 1,016-1,143 and 1,143-1,270 sequences, the mutation rate decreased significantly at the end of the two doses of vaccination (**Figure 4**).

In phase 2 (booster) of vaccination progress, we had four common mutations. E484 and P681 were mutants in 381-508 and 635-762, respectively. D614G and H655 were shown in the 508-635 region. Therefore, by considering the effect of booster vaccination on spike sequence, these three regions that are rated as top high-rate mutations could present as hot spot regions of spike in the second phase of vaccination. Taken together, between the three groups, sequence 762-889 was the most conserved part of spike mutual among groups (**Figures 4C, D**).

### Statistical significance of mutation counts across vaccination rates

The Kruskal–Wallis test revealed differentiated patterns in the distribution of SARS-CoV-2 mutations in response to varying vaccination rates during the initial phase of vaccine rollout. A statistically significant decrease in the incidence of one mutant was identified when comparing populations with a vaccination rate of 20%-50% against those with 70%-100% coverage, yielding a p-value of 0.017. This implies that a decrease in the incidence of single mutations may be linked to increased vaccination coverage, possibly demonstrating the impact of vaccination on reducing the rates of viral mutation.

In a similar vein, the frequency of more than four mutants and more profiles showed significant variation between the 20%-50% and 50%-70% groups, with a p-value of 0.05. This highlights a substantial relationship between intermediate levels of vaccination coverage and the emergence of complex mutations.

The 20%–50% and 50%–70% vaccination rate groups in the “Two Mutant” category, on the other hand, showed a non-significant trend in “Phase 1 (last),” with a p-value of 0.13. Even though this does not reach the threshold for statistical significance, it suggests a potential relationship that needs more research and may become significant with a larger dataset.

**Figure 5:**
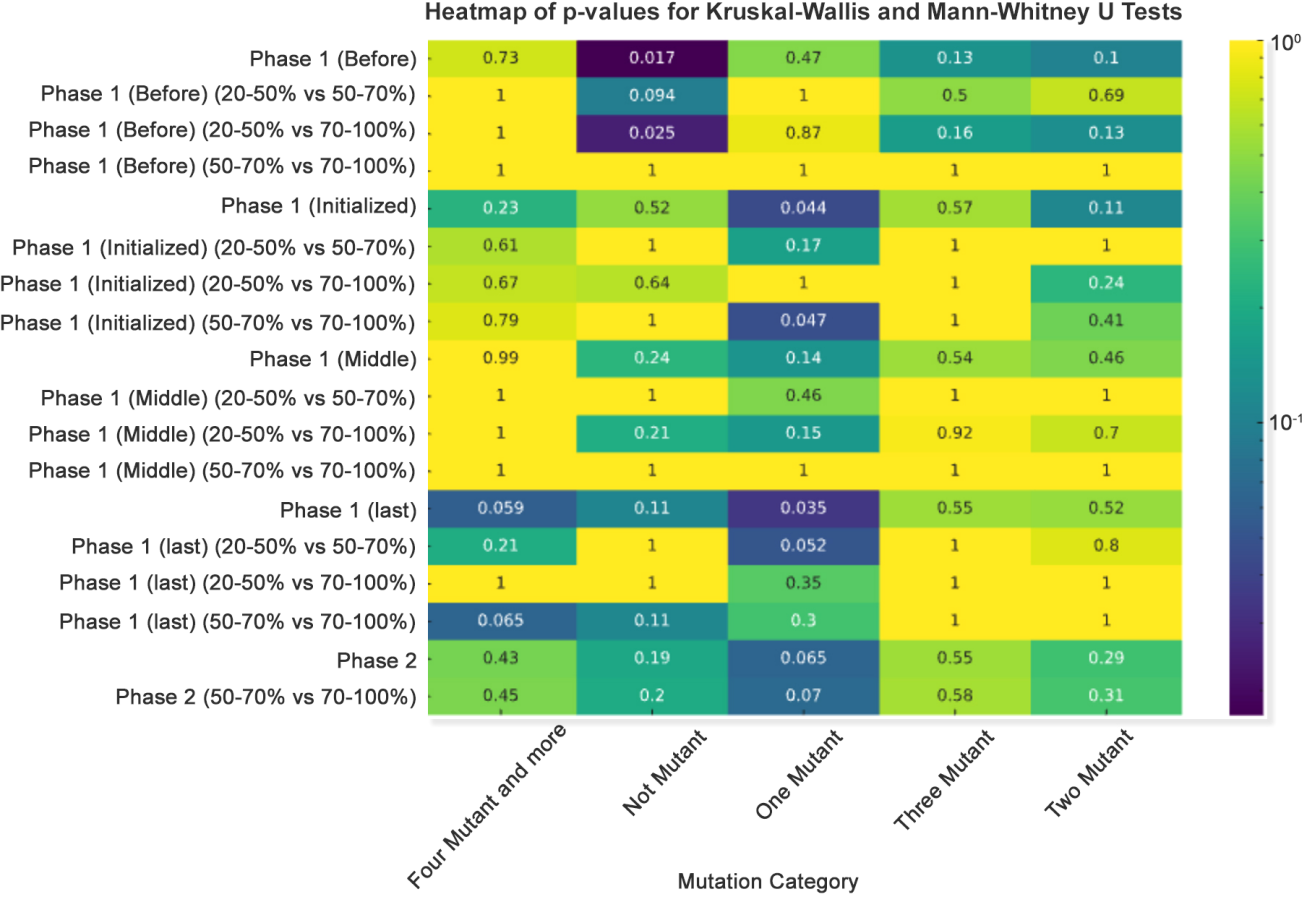
Heatmap of p-values for Kruskal–Wallis and Mann–Whitney U tests. A p-value heatmap was created to illustrate the significance of differences in mutation counts, employing a sequential color scheme to signify p-value gradations, with darker hues indicating lower p-values.

It is noteworthy that the “Not Mutant” category across all phases consistently showed non-significant p-values, indicating no substantial difference in the absence of mutations among the various vaccination rate groups. This consistency implies that vaccination rates may not influence the overall rate of no mutation (**Figure 5**). These results highlight the fact that high vaccination rates exert strong immune selection pressure on SARS-CoV-2, reducing the prevalence of single, random mutations. Instead, the virus is driven to evolve clusters of mutations (e.g., in variants of concern like Delta and Omicron) that enhance immune evasion or transmission efficiency.

**Figure 6:**
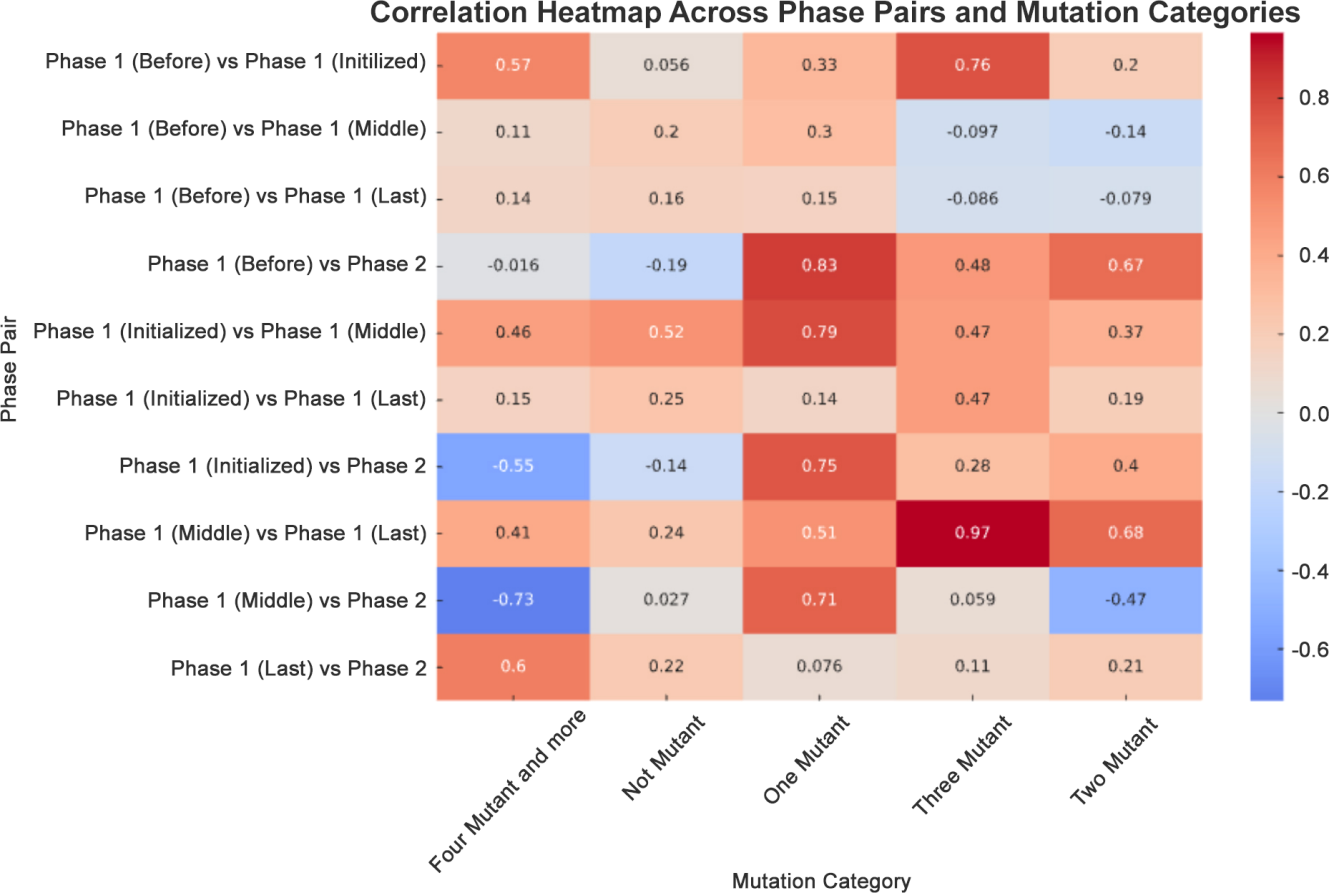
Correlation heatmap across phase pairs and mutation categories. A correlation heatmap was generated to depict the relationship between mutation counts across different phases. This used a diverging color palette to distinguish between positive (blue) and negative (red) correlations.

Correlational analysis across phases demonstrated significant linear relationships in several mutation categories. The correlation coefficient for the “Three Mutant” category between Phase 1 (Before) and Phase 1 (Initialized) was 0.76, denoting a strong positive correlation. Conversely, the correlation between Phase 1 (Middle) and Phase 2 (Booster) for the “Four Mutant and more” category was −0.73, reflecting a strong negative correlation, suggesting an inverse relationship between these phases as the booster campaign unfolded (**Figure 6**).

### Assessment of D614G, E484K, and H655Y mutations on dynamicity and flexibility of spike

To assess how the top mutations examined in this study impact the spike protein’s structure, we utilized the DynaMut website for protein modeling. We determined the variation in vibrational entropy energy *(ΔΔSvibENCoM*) between the wild-type and mutant forms. The results indicate that the mutation at position D614G increased molecular flexibility within the spike structure by 0.768 kcal·mol^−1^·K^−1^, whereas the mutation at position E484K resulted in a flexibility increase of 0.309 kcal·mol^−1^·K^−1^.

**Figure 7:**
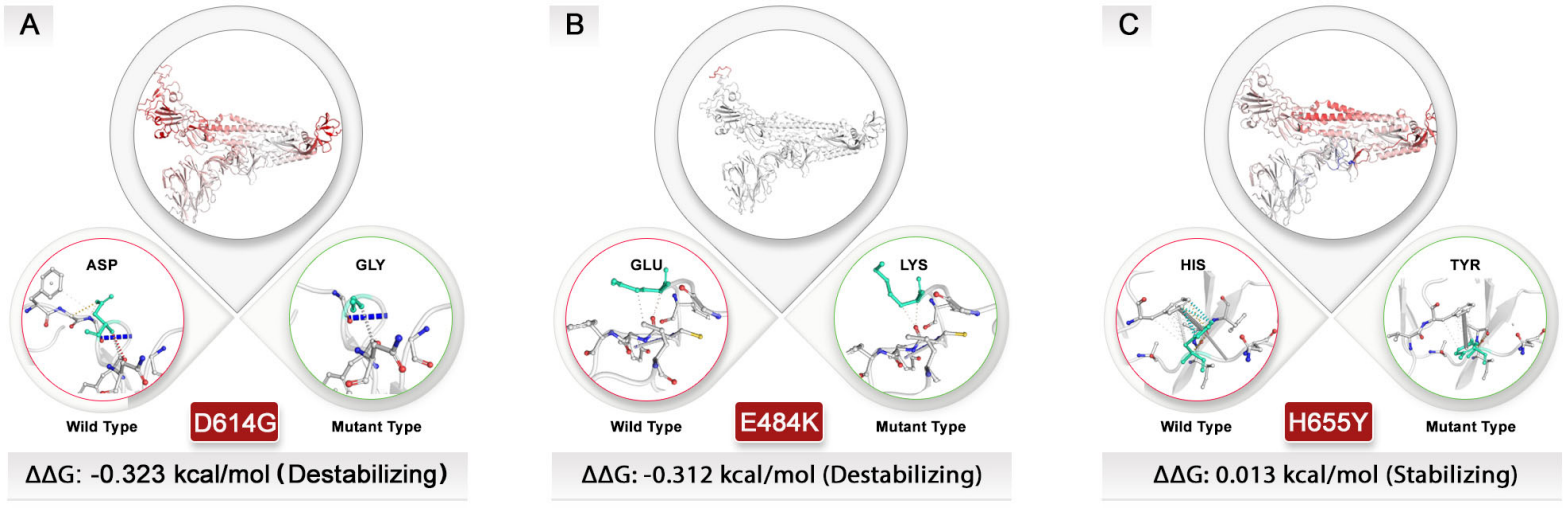
Exploring the structural dynamics of spike protein: unraveling the influence of D614G, E484K, and H655Y mutations. Vibrational entropy changes upon mutation are visually depicted through a color-coded scheme, with amino acids taking on different hues. Notably, the vibrant red color signifies an increase in flexibility within the spike protein. To provide a comprehensive view, wild-type and mutant residues are displayed as light-green sticks, juxtaposed with the neighboring residues engaged in various types of interactions. **(A)** The D614G and **(B)** E484K mutations with negative *ΔΔG* values could destabilize spike structure. **(C)** In contrast, the H655Y mutation had a different effect on the protein’s structure and was stabilized, unlike other top mutations. All dynamic modeling used the 7QUS PDB ID. Due to the unavailability of a PDB ID that presents mutations in the P681 site, modeling for this mutation was not possible.

Conversely, alterations from aspartate to glycine at position 614 and from glutamic acid to lysine at position 484 were found to destabilize the spike protein structure, leading to a binding affinity change of −0.323 and −0.312 kcal/mol, respectively. Among these structural destabilizer mutations, there was also a noteworthy H655Y mutation that offered a slight increase in molecule stabilization with a *ΔΔG* value of 0.013 kcal/mol. However, this mutation simultaneously contributed to an increase in molecular flexibility with a value of 0.117 kcal·mol^−1^·K^−1^. By studying how these amino acid alterations impact intramolecular interactions, we can gain insights into why protein structure destabilization occurs following mutations and vaccination processes (**Figure 7**).

## Discussion

The vaccination strategy is the most effective public health measure to control and alleviate the SARS-CoV-2 pandemic (20, 21). Even after the start of global vaccination, the emergence of new strains, including those resistant to vaccines, remains a major concern. So far, established databases for the SARS-CoV-2 are limited, and their classifications are based on viruses at the genome mutations and evolutionary levels (22, 23). The Sars2Mutant database has facilitated these limitations by providing features such as gene classification, and detection of mutations at protein levels in the various continents, countries, and timelines. Furthermore, this database provides insights into the exact loci of the mutations regarding the frequency in each gene, hotspot, and highly conserved region of SARS-CoV-2 (17). The 762-889 region, as the most conserved part of SARS-CoV-2 in the current study, was shown as part of 16 relatively conservative epitopes (747‐763, 749‐771, 754‐770) including vaccine candidate epitopes (24).

In this regard, the findings of our study showed that with the progress of vaccination, the occurrence of mutations decreases, and at the end of the second-dose vaccination time point, the majority of the sequences showed no mutations. In support of our findings, a SIR-derived model-based study found that the rapid vaccination rate reduces the emergence of vaccine-resistant strains (25). Nevertheless, the percentage of the vaccinated population is not the only factor in the occurrence of mutations and the rise of new strains. The transmission of the virus is another critical factor influencing the emergence of new strains. This could be a plausible explanation for shifting all the study groups to no mutation in their sequences. The study indicates that during the booster vaccination in phase 2, the mutated sequences increased due to increased virus transmission power. This finding is significant because it highlights the need to revise vaccine design to adapt to the evolving genomic variations.

A variety of mutations have been detected in the two phases. However, some of these mutations were abundantly and consistently seen in all groups, meaning that the virus is more prone to keep these mutations. The most dominant reported spike protein mutation detected in all groups is D614G, which enhanced virus replication via increased infectivity and stability (26, 27).

The D614G in Alpha, Delta, Beta, and Omicron variants modulates the binding to the angiotensin-converting enzyme 2 (ACE2) receptor, which could be important in determining virus entry, immune escape, transmissibility increment, and reinfection (28–31). Also, P681 is the other dominant mutation in the spike that could display highly increased fusogenic activity and syncytia formation capability (32). P681 was a mutation common in all groups that increased gradually in the process of vaccination. P681 became one of the dominant mutations in both vaccination phases. P681 mutation cooperates with the furin cleavage site. However, Lubinski et al.’s study demonstrated that this mutation does not affect the proteolytic feature of spike protein and, thereby, the viral entry or cell–cell spread (33). P681 was the mutation only reported in the Delta variant that seems to augment the spike processing and spike fitness compared with the alpha variant (12, 34). Interestingly, this mutation has not been appropriately investigated and detected in isolated human B.1.1.7 samples from USA and India (35).

The data analysis illustrated that E484 was among the common mutations in both two phases of vaccination; however, it had a meaningful increase in the second phase. The E484 site has a functional role in RBD that significantly affects the binding affinity with the ACE2 receptor. Subsequently, a mutation in the E484 site could have a significant impact on the immunogenicity of the RBD protein, which could affect the pathogenicity and transmissibility of the mutant virus (36–38). Aligning the RBD sequence confirmed both Beta and Omicron harboring mutation at residue E484 that were replaced to Lysine for Beta and to Alanine for Omicron variants (39, 40). The bivalent Original/Omicron BA.1 vaccine contains mRNA that carries instructions for cells in the body to make the spike protein that is also on the virus. The cells then make antibodies against the spike protein to help fight off the virus. The bivalent vaccine is effective in producing high levels of neutralizing antibodies against Omicron subvariants (41). Recent research suggests that incorporating more than one strain in vaccine development, booster shots, and other vaccine changes is crucially important (42). Following booster vaccination, the H655Y mutation appeared at a high rate in samples, resulting in increased viral endpoint yields in human respiratory cells, and was associated with enhanced spike processing in the Gamma and Omicron variants. Furthermore, Y655 is found close to the fusion site, enhancing virus–cell fusion. In this regard, vaccination and neutralizing antibodies are the major obstacles in this process. Therefore, this mutation will enable the virus to overcome this problem, potentially giving it an advantage in spreading among populations (43, 44). H655Y, as a notable mutation in the second phase with stabilizing feature for spike protein could be an important for the vaccine industry (45, 46).

An overview of these four prevalent mutations may help us identify key spike structure components, particularly CTD2 and RBD. Given that E484 in RBD, and D614G, H655, and P681 are located in CTD2, these regions of the spike could be considered as the highlighted regions in the COVID-19 vaccine design (47). Vaccination flow alongside appearance and increasing rate of E484 and H655 in the second phase of vaccination by booster dose could explain why these two mutations are resistant to booster dose. The explanation for the outbreak of these variants, which is consistent with earlier research, was provided by the synchronization of the spread of Alpha, Gamma, and significantly Omicron variants with the appearance of these mutations (48).

Higher vaccination rates are associated with a decrease in the incidence of one mutant, which may indicate that immunization limits viral evolution. Conversely, the appearance of more intricate mutation profiles at intermediate vaccination rates—four mutants or higher—may point to a complex interaction between vaccination coverage and virus evolution. The marginal trends observed in some categories, such as the “two mutant” group, highlight areas for further investigation, suggesting that expanding the dataset could unveil additional significant relationships (49, 50).

Our study suggests that mutations in COVID-19 are growing due to the increase in virus transmission capacity, especially after booster vaccination. The study identified four top mutations, two of which were shown in each of the two phases, whereas two high-rate mutations were only reported after booster vaccination in the population. The results highlight the need to revise vaccine design to adapt to the evolving genomic variations by considering conserved spike regions into multi-epitope or universal vaccines. Moreover, vaccine developers can create polyvalent mRNA or protein-subunit vaccines that maintain efficacy even as SARS-CoV-2 continues to evolve. Such a strategy would be helpful to ensure that future vaccine formulations keep pace with the emergence of immune escape mutations while reducing the need for frequent vaccine reformulation.

## Data Availability

The original contributions presented in the study are included in the article/**Supplementary Material**. Further inquiries can be directed to the corresponding authors.
