# Supplementary material for: Intersecting SARS-CoV-2 spike mutations and global vaccine efficacy against COVID-19
Source: Front Immunol. 2025 Mar 7;16:1435873. doi: 10.3389/fimmu.2025.1435873 (PMC11925781; doi:10.3389/fimmu.2025.1435873)
Supplement: Supplementary file 1 [file Table1.docx]

Supplementary Material

**Intersecting SARS-CoV-2 Spike Mutations & Global Vaccine Efficacy Against COVID-19**

Samaneh Tokhanbigli^1+^, Samira Salami Ghaleh^2, +^, Karim Rahimian^3, +^, Mohammadamin Mahmanzar^4^ , Saleha Bayat ^5^, Shahrzad Ahangarzadeh^6^, Bahman Moradi^7^, Reza Mahmanzar^8,^ Yunliang Wang^9*^, Brian G. Oliver^10,11, *^,[Youping Deng](https://pubmed.ncbi.nlm.nih.gov/?size=200&term=Deng+Y&cauthor_id=34582894) ^4, *^

1. Discipline of Pharmacy, Graduate School of Health, University of Technology Sydney, Australia ([tokhanbigli.s@gmail.com](mailto:tokhanbigli.s@gmail.com))
2. Department of Computer Science, University of Tabriz, Tabriz, Iran ([samira.salami80@gmail.com](mailto:samira.salami80@gmail.com))
3. Institute of Biochemistry and Biophysics (IBB), University of Tehran, Tehran, Iran, ([karim.rahimian@gmail.com](mailto:karim.rahimian@gmail.com))
4. Department of Quantitative Health Sciences, John A. Burns school of medicine, University of Hawaii at Manoa, Honolulu, hi 96813, ([mahmanza@hawaii.edu](mailto:mahmanza@hawaii.edu))
5. Department of Biology & Research Center for Animal Development Applied Biology, Mashhad Branch, Islamic Azad University, Mashhad, Iran , ([saba80012@gmail.com](mailto:saba80012@gmail.com))
6. Infectious Diseases and Tropical Medicine Research Center, Isfahan University of

Medical Sciences, Isfahan, Iran ([shahrzadahangar@yahoo.com](mailto:shahrzadahangar@yahoo.com))

1. Department of Biology, Faculty of Sciences, Shahid Bahonar University of Kerman, Kerman, Iran ([bahmanmoradi.biocan1400@gmail.com](mailto:bahmanmoradi.biocan1400@gmail.com))
2. Department of Biology, Science and Research Branch, Islamic Azad University, Tehran Iran ([reza.mahmanzar.70@gmail.com](mailto:reza.mahmanzar.70@gmail.com))
3. Department of Neurology, the Second Affiliated Hospital, Zhengzhou University, Zhengzhou, 450014, Henan, China. ([wangyunliang81@163.com](mailto:wangyunliang81@163.com) )
4. School of Life Sciences, Faculty of Science, University of Technology Sydney, Sydney, Australia ([brian.oliver@uts.edu.au](mailto:brian.oliver@uts.edu.au) )
5. Respiratory Cellular and Molecular Biology, Woolcock Institute of Medical Research, nsw 2037, Australia, ([brian.oliver@uts.edu.au](mailto:brian.oliver@uts.edu.au))

+ These authors have the same contribution.

*Corresponding Authors:

### *[Youping Deng](https://pubmed.ncbi.nlm.nih.gov/?size=200&term=Deng+Y&cauthor_id=34582894), PhD.

### Department of Quantitative Health Sciences, John A. Burns School of Medicine, University of Hawaiʻi at Mānoa,

### **Tel:** +1808.692.1664 (Office)

### **Fax:** 808.692.1970

### **Email:** [dengy@hawaii.edu](mailto:dengy@hawaii.edu)

* Brian G. Oliver, PhD.

- School of Life Sciences, Faculty of Science, University of Technology Sydney, Sydney, Australia
- Respiratory Cellular and Molecular Biology, Woolcock Institute of Medical Research, NSW 2037, Australia,

**Tel:** +61 2 9114 0367;

**Email:** [Brian.Oliver@uts.edu.au](mailto:Brian.Oliver@uts.edu.au)

* Yunliang Wang, PhD.

### Department of Neurology, the Second Affiliated Hospital, Zhengzhou University, Zhengzhou, 450014, Henan, China. [wangyunliang81@163.com](mailto:wangyunliang81@163.com).

**Email:** [wangyunliang81@163.com](mailto:wangyunliang81@163.com)

Before the vaccination, arginine was the substituted A.A in the P681 mutation. With the progress of immunization at the initial and middle of vaccination, histidine was the replaced A.A with proline in P681; however, in the end, P681 was deleted in the investigated samples of this group (Fig 1A).

P681H/R/- is the only mutation detected in all the stages of the vaccination time points. P681 mutation substituted with A.A histidine was seen before, initial, and middle with a gradual increase (11.7, 42.94, 48.66 %). P681mutation with A.A conversion to R was observed during the initial, middle, and end with 12.1, 11.92, and 26.42%, respectively. The proline deletion at the 681 positions was the dominant substitution at the end of the selected vaccination time point (Fig 1B).

Like the other groups P681H/R/- among the mutations observed in the initial, middle, and end of the designated time points for a vaccination with (64.1, 94.43, and 99.23%) respectively. P681 mutation with A.A conversion to histidine was dominant during the initial (58.07 %) and middle (88.1 %); however, the deletion of the proline at the 681 positions was the prevalent substitution seen at the end of the selected vaccination time point with 78.73 % (Fig 1C).


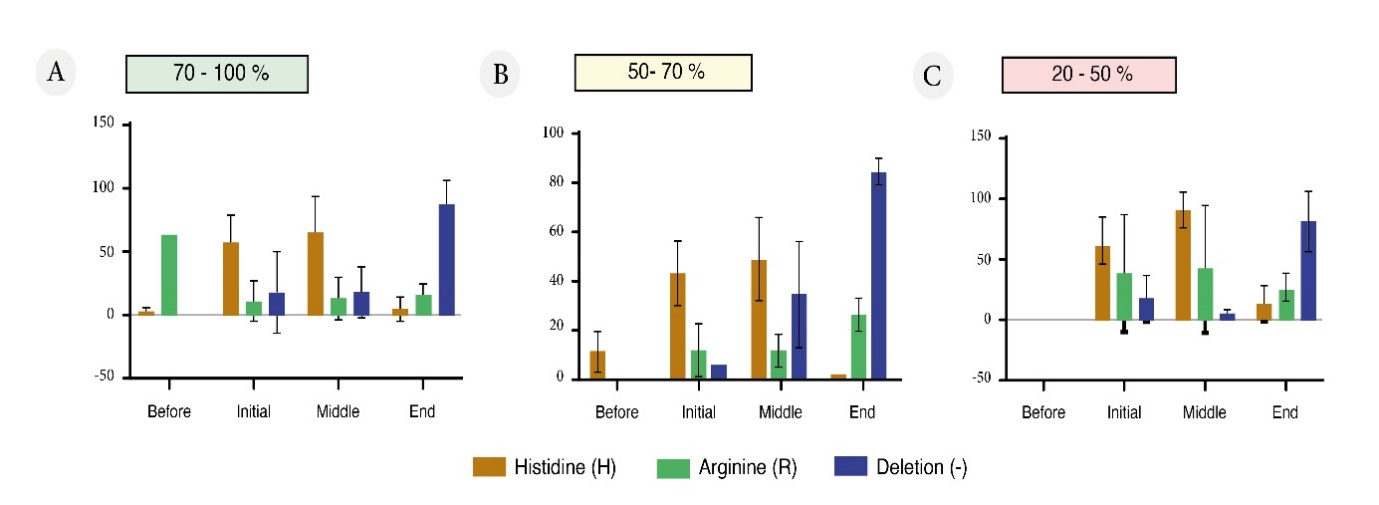


**Supplementary figure 1. The amino acid substitution of P681 mutation during the vaccination with different immunized populations.**

(A) In the group with a 70-100% vaccinated population, with vaccination progress, P681- mutation was dominant at the selected time point at the end of vaccination. (B) In the group with a 50-70% vaccinated population, P681 deletion was dominant at the selected time point at the end of vaccination with the progress of vaccination. (C) In the group with a 20-50% vaccinated population, the conversion of proline to histidine and arginine was the prevalent substitution in this group's initial and middle vaccination. Like the other groups, P681 deletion was dominant at the selected time point at the end of vaccination.
